# Identification of Cellular Calcium Binding Protein Calmodulin as a Regulator of Rotavirus A Infection during Comparative Proteomic Study

**DOI:** 10.1371/journal.pone.0056655

**Published:** 2013-02-20

**Authors:** Shiladitya Chattopadhyay, Trayambak Basak, Mukti Kant Nayak, Gourav Bhardwaj, Anupam Mukherjee, Rahul Bhowmick, Shantanu Sengupta, Oishee Chakrabarti, Nabendu S. Chatterjee, Mamta Chawla-Sarkar

**Affiliations:** 1 Division of Virology, National Institute of Cholera and Enteric Diseases, Kolkata, West Bengal, India; 2 Genomics and Molecular Medicine, CSIR-Institute of Genomics and Integrative Biology, Delhi, India; 3 Department of Zoology, University of Calcutta, Kolkata, West Bengal, India; 4 Structural Genomics Section, Saha Institute of Nuclear Physics, Kolkata, West Bengal, India; 5 Division of Biochemistry, National Institute of Cholera and Enteric Diseases, Kolkata, West Bengal, India; University of Hong Kong, Hong Kong

## Abstract

Rotavirus (RV) being the major diarrhoegenic virus causes around 527000 children death (<5years age) worldwide. In cellular environment, viruses constantly adapt and modulate to survive and replicate while the host cell also responds to combat the situation and this results in the differential regulation of cellular proteins. To identify the virus induced differential expression of proteins, 2D-DIGE (Two-dimensional Difference Gel Electrophoresis) based proteomics was used. For this, HT-29 cells were infected with RV strain SA11 for 0 hours, 3 hours and 9 hours post infection (hpi), differentially expressed spots were excised from the gel and identified using MALDI-TOF/TOF mass spectrometry. 2D-DIGE based proteomics study identified 32 differentially modulated proteins, of which 22 were unique. Some of these were validated in HT-29 cell line and in BALB/c mice model. One of the modulated cellular proteins, calmodulin (CaM) was found to directly interact with RV protein VP6 in the presence of Ca^2+^. Ca^2+^-CaM/VP6 interaction positively regulates RV propagation since both CaM inhibitor (W-7) and Ca^2+^ chelator (BAPTA-AM) resulted in decreased viral titers. This study not only identifies differentially modulated cellular proteins upon infection with rotavirus in 2D-DIGE but also confirmed positive engagement of cellular Ca^2+^/CaM during viral pathogenesis.

## Introduction

Viruses constantly adapt to and modulate the host environment during replication and propagation. Both DNA and RNA viruses encode multifunctional proteins that interact with and modify host cell proteins. While viral genomes were the first complete sequences known, the corresponding proteomes are being elucidated now. Even more daunting is the task to globally monitor the impact of viral infection on the proteome of the host cell because of the dynamic nature of proteins, including post-translational modifications, enzymatic cleavage and activation or destruction by proteolytic events.

Rotavirus (RV) which belongs to the genus Reoviridae, causes an estimated 527,000 diarrheal deaths each year, with >85% of these deaths occurring in children aged below five years in low-income countries of Africa and Asia [1]. RV contains eleven double stranded RNA as genome which encodes twelve proteins. Six of the twelve proteins are nonstructural (NSP1-NSP6), i.e. these are expressed only inside host cells and the other six form integral part of the virus core and surface, hence are known as structural proteins (VP1-VP4, VP6 & VP7) [2], [3]. A few studies have addressed the issue of the molecular mechanism of how host cells might respond to rotavirus infection. Rotavirus infection elicits production of cytokines IL-8 and RANTES and GRO-α [4]. Human intestinal Caco-2 cells infected with either RV strains Wa (human) or SA-11(Simian), induced the expression of COX-2 mRNA and secreted PGE2 [5]. c-Jun NH2-terminal kinase (JNK) and c-Jun (component of AP-1), which are upstream to NF-κB and AP-1 signaling were activated on infection with RRV in HT-29, Caco-2, and MA104 cells [6]. Activation of p38 during RRV infection was also observed in Caco-2 and MA104 cells but not in HT-29 cells. Infection of rotavirus has been found to induce expression of cellular Hsp90 and Akt [7]. Rotavirus induces expression of IFN stimulated genes (ISGs) contrarily it also prevents nuclear translocation of STAT1 and STAT2, resulting in inhibition of ISG induction by IFNs [8], [9]. Furthermore rotavirus NSP1 protein can induce proteasome-mediated degradation of IRF3, IRF5, and IRF7 to subvert induction of IFN-β [10]. NSP1 has also been shown to induce proteasome-mediated degradation of β-TrCP, resulting in stabilization of IκB & repression of NFκB [11].

Though few studies based on microarray and other techniques have analyzed cellular effects during RV infection, large scale proteome analysis studies are not well documented. Cuadras *et al.* described time dependent transcriptome level analysis of RV (RRV strain) infection in Caco-2 cells at 1 hpi, 6 hpi, 12 hpi & 24 hpi where major changes were observed at 12 hpi or more hpi [12]. Comparative transcriptome analysis with different RV strains SA11, Wa & A5–13 revealed that though strain specific differences are there, 131 genes were commonly induced by all three strains [13]. The first 2D gel electrophoresis and MS/MS based study of rotavirus was reported by Aimin Xu *et.al.,*which demonstrated differential expression of proteins in mock and RV-infected MA104 cells by 2D gel electrophoresis [14]. Four host proteins were upregulated during infection, of which two were identified as members of glucose regulated chaperone family namely GRP78 (BiP) and GRP94 (endoplasmin) which locate to endoplasmic reticulum, a site of RV morphogenesis. Several members of the class of ER-localized molecular chaperones were also shown to be altered by enterotoxin NSP4 in a proteomics based study [15]. Recently, Zambrano *et.al*. reported two-dimensional difference gel electrophoresis (2D-DIGE) based proteomic change induced by RV OSU strain, focused only on interferon response [16]. Inspite of these previous studies, the overall effect of RV on host cell protein remain elusive.

This study was initiated to identify differentially regulated proteins both during early and late infection in RV infected cells. Results of the 2D-DIGE followed by MALDI-TOF/TOF mass spectrometry 2D-DIGE followed by MALDI-TOF/TOF mass spectrometry revealed large number of differentially modulated proteins following RV infection. Some of the identified proteins such as Calmodulin (CaM) were further characterized to understand their role during infection. CaM was upregulated during early hour of infection and it was found to interact with RV protein VP6 in a Ca^2+^ dependent manner.

## Materials and Methods

### Ethics Statement

The animal investigation was approved by the Institutional Animal Ethics Committee, National Institute of Cholera & Enteric Diseases, Indian Council of Medical Research (Registration No.- NICED/CPCSEA/AW/(215)/2012-IAEC/SSO) and (Approval # 65/20/08/2009), registered under “Committee for the Purpose of Control and Supervision of Experiments on Laboratory Animals,” Ministry of Environment and Forests, Government of India and conforms with the Guide for the Care and Use of Laboratory Animals published by the United States National Institutes of Health Publication 85–23, revised 1996.

### Cell Culture and Virus Infection

The human colon carcinoma cell, HT-29 (ATCC number: HTB-38™) and human embryonic kidey cell line, 293 (ATCC number: CRL-1573™) were cultured in DMEM supplemented with 10% FBS (Invitrogen, USA) and 1% antibiotic-antimycotic solution (Invitrogen, USA) and kept in a 5% CO_2_ incubator at 37°C until optimum growth was observed. MA104 (ATCC number: CRL-2378™) cells were cultured in MEM instead of DMEM. The cell culture adapted RV strain SA11-H96 (simian) was added to the cells at the multiplicity of infection (moi) of 1, 3 and 5; after observing the immunofluorescence microscopy of HT-29 cells (Figure S1), moi 3 was chosen for further experiments and infected as described previously for required hours post infection (hpi) [17]. For 2D-DIGE experiments, virus infected cells at 0 hpi, 3 hpi and 9 hpi were lysed in buffer containing 7 M urea, 2 M thiourea, 30 mM Tris-Cl (pH 8.5), 1× Protease inhibitor cocktail (Sigma Aldrich, USA) and 1× Phosphatase inhibitor cocktail (Sigma Aldrich, USA) by sonicating at 30 kHz followed by centrifugation at 13200 g for 15 mins at 4°C. Protein concentration was measured by using Bradford method (Sigma Aldrich, USA) [18].

### Plaque Assay

For calculating viral titers, plaque assays were performed according to previously described protocol [7], [17]. Viral PFU was then calculated as PFU/ml (of original stock)  = 1/dilution factor x number of plaques x 1/(ml of inoculum/plate) [19].

### Two-dimensional Difference Gel Electrophoresis [2D-DIGE]

**Figure 1:**
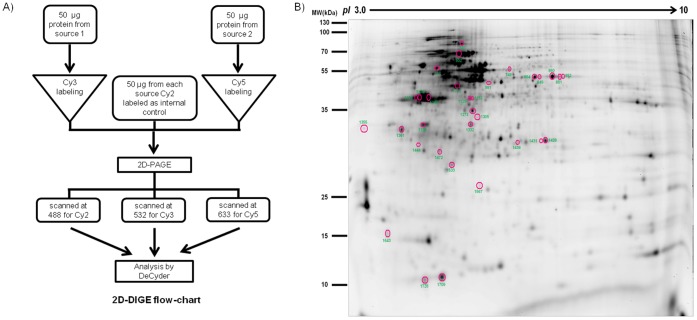
2D-DIGE sample preparation flowchart & preparatory gel image. A. Schematic diagram of crucial steps in 2D-DIGE sample preparation. **B.** Preparatory Gel image showing differentially expressed protein spots. The spots are circled along with their corresponding spot numbers (Master/Spot IDs).

2D-DIGE was performed to identify proteins that are differentially expressed in 0 hpi, 3 hpi and 9 hpi using a loop design approach as described earlier, which enables comparison between three groups in a DIGE experiment [20]. Any two groups were compared based on two biological and two technical replicates (dye swaps were done to account for dye biasness). Samples for each time point were pooled from two different replicates making every time point a mixture of two different samples. Fifty micrograms of each sample was labeled with either 400 pmol of Cy3 or Cy5 (GE Healthcare, NJ). An internal standard was created by pooling 50 µg of each of the six samples which was labeled with Cy2. The samples were incubated with the dyes for 30 minutes in the dark for labeling and the reaction was stopped by adding 10 mM lysine. Samples were then reduced, denatured in rehydration buffer followed by IEF and 2D gel electrophoresis as described previously [21]. For preparatory gel, 1 mg of unlabeled protein obtained from the pool was used. A flow-chart of the sample processing for 2D-DIGE is schematically presented here (Fig. 1A).

### Image Acquisition and Analysis

The gels were scanned at 488 nm (Cy2), 532 nm (Cy3), and 633 nm (Cy5) using Typhoon Trio variable mode imager (GE Healthcare, NJ) and the images were analyzed by DeCyder 2Dsoftware (version 6.5, GE Healthcare, NJ) using Differential In gel Analysis module (DIA) and Biological Variation Analysis (BVA) module. The coomassie stained preparatory gel was scanned with Cy5 excitation laser with a resolution of 100 µm. Protein spots exhibiting a statistically significant (p<0.05) difference in intensity between the three experimental groups were excised from the preparatory gel and identified using MALDI-TOF/TOF mass spectrometry [21].

### MALDI TOF/TOF Analysis

The differentially expressed spots were digested with trypsin as mentioned earlier [21], [22]. The digested and extracted peptides were spotted in triplicates onto MALDI sample plate and mixed with equal volume of α-cyano-4- hydroxyl-cinnamic acid matrix solution (10 mg/ml) in 0.1% TFA and 50% ACN. Peptide mass spectra were obtained using a MALDI-TOF/TOF 5800 mass spectrometer (AB Sciex, CA) operating in reflectron mode. Laser intensity was set at 3200 with 1000 total shots over the window of m/z 800 to m/z 4000. MS peptide fingerprint and MS/MS peptide sequencing search was performed in Swissprot (51.6) database (257964 sequences; 93947433 residues) using the ProteinPilotTM software (version 4.0) via Mascot search engine (version 2.2, AB Sciex, CA). Tryptic digestion with a maximum of 1 missed cleavage was considered. All cysteines were considered modified with carbamidomethylation (+57.012) and a variable modification of methionine oxidation (+15.9949) was also taken into account. The monoisotopic precursor ion tolerance was set to 100 ppm and the MS/MS ion tolerance to 0.6 Da. Protein identifications were accepted with a statistically significant probability based Mowse score (p≤0.05). Proteins with at least 2 peptides that were unique to the specific protein were only considered.

### Western Blotting Validation of Identified Proteins in Cell Culture

Confluent HT-29 cells were infected with SA11 strain with moi of 3 and lysed with buffer at appropriate time point. Twenty-five micrograms of protein was run on 12% SDS-PAGE (sodium dodecyl sulfate polyacrylamide gel electrophoresis) and western blotting was performed according to standard protocol. The primary antibodies (Santa Cruz Biotechnology,Inc, USA ) for cellular proteins and anti-RV primary antibody of VP6 (HyTest Ltd., Finland) followed by HRP conjugated secondary antibodies (Thermo Scientific, USA) were used to detect protein using Immobilon Western Chemiluminescent HRP substrate (Millipore, USA) and imaged onto BioMax MR Film (Kodak). GAPDH was taken as internal control for all experiments.

Independent biological triplicate samples were run separately in SDS-PAGE and later immunoblotted. Independent triplicate immunoblots were scanned and quantitated using Quantity-one software version 4.6.3 (Bio-Rad, Hercules, CA) in a GelDoc XR system [17], [23].

### Quantitative Real-time Reverse Transcription-PCR (qRT-PCR)

HT-29 cells were infected as described before and RNA was prepared using Trizol reagent (Ambion Inc., USA) and dissolved in RNase-free water. Two micrograms of total RNA from each set of sample were reverse transcribed with SSII RT (Invitrogen, USA). Real-time PCR were performed in triplicates using the SyBr green (Applied Biosystems, USA) in a Step One plus Real-Time PCR system (Applied Biosystems, USA) taking 18S rRNA as an internal control. Fold change for gene expression was calculated using the formula 2^−ΔΔCt^ [24].

### Western Blotting Validation of Protein Expression in BALB/c mice Model

To investigate and validate the results, a RV intestinal ligated-loop model was established in BALB/c mice. BALB/c mice of 4 weeks age were anesthetized using isoflurane (Sigma Aldrich, USA) in a closed chamber [25], [26]. An incision was made through its skin to take out the intestine. Approximately 2.5 cm of lower small intestine (ileum) was tied without hampering the normal blood flow of intestine. Trypsin-activated 150 µl of RV strain SA11 was injected into each of the loops. Two BALB/c mice were kept mock infected as an experimental control. After 6 hr, 12 h and 16 hr post inoculation, mice were checked for accumulation of water and sacrificed according to previously described method [25]–[28]. Ligated-loop was taken out and washed with sterile 1×PBS. Mice intestinal loop was homogenized in modified RIPA buffer and immunoblotted as described before.

### Confocal and Immunofluorescence Microscopy

MA104 cells were grown in four-welled chambered slides (BD Pharmingen, USA) and infected with RV strain SA11 at different hpi. The cells were fixed at 0 hpi, 3 hpi and 9 hpi with 4% paraformaldehyde (w/v in PBS) for 10 mins at room temperature and permeabilized with 0.1% Triton-X-100 for 15 mins at 4°C. The remaining protocol was followed as described previously [29], [30]. The slides were examined either under a confocal or a fluorescence microscope (Carl Zeiss, Gottingen, Germany).

### Co-immunoprecipitation

Co-immunoprecipitation (Co-IP) was performed using Pierce Co-IP kit according to manufacturer’s instruction. Either anti-VP6 or CaM antibody was used to detect the prey protein in western blotting depending on the bait antibody used [31]. A five percent volume of lysate from each set was kept into sample tube separately as input to run in gel.

### Plasmid Construction and Transfection

Full-length NSP3 and VP6 from RV-SA11 which has been cloned previously into the pCDNA6 (Invitrogen, USA) in our lab were used here [29]. The vectors were transfected into 293 cells using the Lipofectamine 2000 tranfection reagent (Invitrogen, USA) according to the manufacturer's instructions.

### *In-vitro* Coupled Transcription-translation

Plasmids (pCDNA 6.1) encoding the full length VP6 under the T7 promoter was subjected to *in vitro* coupled transcription-translation using TNT Quick Coupled Transcription/Translation System (Promega, USA) according to the manufacturer’s specifications. Briefly, 2 µg of circular plasmid was added to the TNT Quick Master Mix and incubated in the presence of Transcend biotin-lysyl-tRNA (Promega, USA) in a 50 µl reaction volume for 50–90 min at 30°C and the products were separated by SDS PAGE and immunoblotted using Pierce High sensitivity streptavidin-HRP (Thermo Scientifics, Rockford, USA) [29]. Recombinant proteins were purified on Ni^2+^-NTA magnetic agarose beads under native conditions and the purity was validated by immunoblot analysis using antibodies against VP6. The purified protein was subjected to co-immunoprecipitation using purified calmodulin protein (Merck Millipore, USA). Co-IP was performed according to previously described protocol in CHAPS Co-IP buffer. 1 mM CaCl_2_ or 1 mM CaCl_2_ with 1 mM EGTA (Ca^2+^ chelator) was added in Co-IP buffer because interaction could be Ca^2+^ dependent or Ca^2+^ independent.

### Identification of Putative CaM Binding Site

Putative CaM binding site was predicted in CaM target database (http://calcium.uhnres.utoronto.ca/ctdb/pub_pages/search/index.htm) using the protein sequence of VP6 gene of RV strain SA11 (Accession number AEI91047). Result output is shown with numerical value where series of 9 means highly significant position and the value reduces gradually with decrease in significance [32]. X-Ray crystallographic structure of RV-VP6 monomer was downloaded from (http://www.rcsb.org/pdb/explore.do?structureId=1QHD) and binding sequence analysis was done by Pymol (V 1.01). Trimeric 3-D reconstructive structure of RV-VP6 was taken from (http://pdbj.org/emnavi/emnavi_detail.php?id=1752) and binding site was analyzed by UCSF-Chimera.

### Proteomic Level Confirmation of CaM-VP6 Interaction

To confirm interaction of CaM and VP6, RV infected HT-29 cell lysates (0 hpi & 3 hpi) were coimmunoprecipitated using anti CAM antibody. Immunoprecipitates were later analysed by SDS-PAGE. A protein band with molecular weight of approximately 45 kDa, present only at 3 hpi but not at 0 hpi identified as probable VP6 protein, was excised from the gel, digested and analyzed by MALDI TOF/TOF. VP6 protein sequence was trypsin digested by online digestion tool PeptideMass (http://web.expasy.org/peptide_mass/) and manually matched with peptide masses found in MALDI TOF/TOF analysis. Manually matched peptide masses were then searched in Mascot Peptide Mass Fingerprint tool. All modifications and conditions were considered as described previously in this study.

### Calcium Chelation and W7 Treatment

To investigate the calcium dependence of CaM VP6 interaction, one set of cells were treated with 75 µM BAPTA-AM (1,2-bis-(o-Aminophenoxy)-ethane-N,N,N',N'-tetraacetic acid, tetraacetoxymethyl ester) in DMSO (Dimethyl sulfoxide) which was added post-adsorption of virus and kept for 3 hrs to chelate intracellular Ca^2+^ [33], [34]; in parallel another set was prepared by treating with DMSO as a negative control. To monitor the efficiency of chelation by BAPTA-AM, intracellular [Ca^2+^] was measured with Fura2-AM as described before [35].

Cytotoxicity of Fura2-AM was tested using MTT assay (data not shown) [36]. HT-29 cells were treated with increasing concentration (0–50 µM) of W-7 (in DMSO), a CaM antagonist and after 12 h, cell viability was measured (data not shown). The assay suggested 90–98% viability of cells at 30 µM, thus this concentration was used for experiments in this study. Confluent HT-29 cells were infected with RV strain SA11 (moi 3) for different hpi. After adsorption, W-7 (in DMSO) was added at 30 µM concentration or mock treated with DMSO as a negative control. Cells were lysed either at 3 hpi or at different time-points. Effect of W-7 or BAPTA-AM on RV infection was analyzed by subjecting cell lysates to Co-IP or immunoblotting experiments.

### Statistical Analysis

2D-DIGE experimental data was statistically tested using t-test by DeCyder software (version 6.5). A non-parametric test (Mann-Whitney Test) has been performed taking the spot abundances of identified proteins. For other experiments mean ± standard error (S.E.) of at least three independent biological replicates (n≥3) was considered for analysis. In all tests, P≤0.05 was considered statistically significant.

## Results

### 2D-DIGE Profiling of RV SA11 Infected HT29 Cells

**Figure 2:**
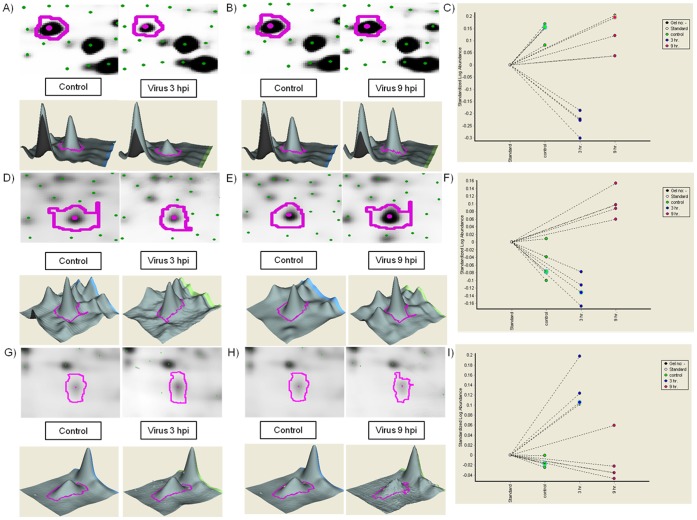
Differential expression of ATP synthase subunit β, inorganic pyrophosphatase and calmodulin. **A** and **B** shows images of ATP synthase subunit β (spot no. 737) in control and 3 hpi or 9 hpi RV infected samples respectively. **D** and **E** shows images of Inorganic Pyrophosphatase (spot no. 1273) in control and 3 hpi or 9 hpi RV infected samples respectively. **G** and **H** shows images of Calmodulin (spot no. 1643) in control and 3 hpi or 9 hpi RV infected samples respectively. **C**, **F** and **I** show the Standard Log Abundance plot in standard, control and treated sets of all replicate gels respectively for ATP synthase subunit β, inorganic pyrophosphatase and calmodulin.

Differentially regulated host proteins during RV infection were analyzed by 2D-DIGE. Two different time points (3 hpi and 9 hpi) were compared with each other and with 0 hpi (control). Samples of each time point were pooled from two different sets. The entire data was highly statistically significant (p<0.05) in all biological as well as technical replicates. Result of Mann-Whitney test suggested that the data was still significant for example the spot abundances of CaM at 3 hpi were significantly different from those at 0 h (p = 0.0286). Cy2 staining for internal control was consistent in each set of gels. Analysis using the Swissprot and MSDB database using the MASCOT search engine resulted in identification of total 32 differentially expressed protein spots (as seen in Fig. 1B) of which 22 were unique. Representative protein spots of ATP synthase subunit β (Fig. 2A, B & C), inorganic pyrophosphatase (Fig. 2D, E & F) and calmodulin (Fig. 2 G, H & I) were shown. Some of the spots that had different pI and hence were resolved as distinct spots in the 2D gel, were of the same protein as revealed by MS analysis. This could be due to either degradation or cleavage of the proteins or various post translational modifications. Out of the 22 unique proteins, 7 were downregulated and 4 were upregulated at 3 hpi. At 9 hpi, among unique proteins 3 were downregulated and 9 were upregulated. Proteins which were identified multiple times in the procedure revealed an interesting profile. As expected, RV structural protein VP6 was the most differentially regulated protein showing 5.8-fold upregulation at 9 hpi, confirming efficient virus infection of cells. Among cellular proteins acidic leucine-rich nuclear phosphoprotein 32 family member A was upregulated 1.3-fold at 3 hpi and 2.18-fold at 9 hpi, whereas mitochondrial ATP synthase subunit beta was significantly downregulated (2.36-fold) at 3 hpi. The proteins which were differentially modulated at any or both of the time points are shown in Table 1.

**Table 1:** List of differentially modulated proteins during GARV SA11 infection in HT-29 cells.

AccessionNo.	Master ID	Av ratio C vs 3 hr	p-value C vs 3 hr	Av ratio C vs 9 hr	p-value C vs 9 hr	MOWSE Score	No. of Peptide	Sequence Coverage (%)	ppm Error	MW	pI	Protein Name
P38646	310	−1.83	0.0044	−1.2	0.2	314	8	23	80	73635	5.87	Stress-70 protein
P11142	502	−1.37	0.004	1.11	0.18	561	12	39	82	70854	5.37	Heat shock 70 kDa protein 8
P06576	737	−2.36	1.90E+05	1.01	1	556	12	48	52	56525	5.26	ATP synthase subunit beta
P06576	748	−1.4	0.0024	−1.28	0.029	228	10	37	25	56525	5.26	ATP synthase subunit beta
P06733	849	−1.13	0.08	1.35	0.0061	132	4	41	16	47139	7.01	Enolase
P06733	851	−1.2	0.017	1.46	0.00017	316	10	39	25	47139	7.01	Enolase
P06733	852	−1.22	0.019	1.53	0.0014	369	11	17	44	47139	7.01	Enolase
P06733	860	−1.28	0.00038	1.5	0.00011	338	7	43	54	47139	7.01	Enolase
P06733	864	−1.19	0.032	1.33	0.022	683	13	43	68	47139	7.01	Enolase
P03531	981	−1.08	0.99	5.8	0.0013	264	4	20	67	44845	5.81	VP6 protein (Major capsid protein)- Simian 11 rotavirus A
P06748	1123	1.32	0.00025	1.22	2.70E+05	146	7	37	22	32726	4.64	Nucleophosmin (NPM)
P55884	1125	−1.25	0.0027	1.03	0.44	90	3	40	65	36479	5.38	Eukaryotic translation
												initiation factor 3 subunit 2
P06748	1130	1.25	0.00097	−1.04	0.36	169	6	39	89	32555	4.64	Nucleophosmin (NPM)
P06748	1149	1.38	2.80E+05	1	0.99	288	9	36	91	32555	4.64	Nucleophosmin (NPM)
O94760	1152	−1.06	0.25	1.28	0.029	92	5	30	46	31102	5.53	NG,NG-dimethylarginine
												dimethylaminohydrolase 1
Q15181	1273	−1.18	0.06	1.42	0.0027	142	6	31	82	32639	5.54	Inorganic pyrophosphatase
P47756	1305	−1.29	0.088	1.49	0.02	121	4	35	82	31331	5.36	F-actin capping protein subunit beta
P35232	1332	1.06	0.3	−1.26	0.0024	309	8	42	14	29843	5.57	Prohibitin
P06753	1336	1.22	0.0058	1.42	0.0003	96	3	36	40	32856	4.68	Tropomyosin
P39687	1355	1.3	0.17	2.18	0.0042	169	5	31	80	28568	3.99	Acidic leucine-rich nuclear
												phosphoprotein 32 family
												member A
P24534	1361	−1.13	0.17	1.38	0.0021	142	5	32	17	24919	4.5	Elongation factor 1-beta
P60174	1428	−1.11	0.0011	1.62	4.40E+06	397	7	52	93	26653	6.45	Triosephosphate isomerase
P60174	1431	−1.16	0.026	1.52	0.00027	353	7	52	93	26653	6.45	Triosephosphate isomerase
P32119	1438	−1.39	0.001	1.1	0.22	381	8	44	84	21878	5.66	Peroxiredoxin
P60174	1444	−1.24	0.0073	1.14	0.069	115	3	48	18	26653	6.45	Triosephosphate isomerase
P21964	1472	−1.21	0.00036	1.43	0.00072	101	3	62	84	30018	5.26	Catechol O-methyltransferase
O75947	1533	1.3	0.00039	−1.01	7.10E−01	75	4	48	9	18537	5.21	ATP synthase D chain
P08294	1567	−1.15	0.052	1.45	0.00034	131	3	59	70	15926	5.7	Superoxide dismutase (Cu-Zn)
P62158	1643	1.39	0.00083	1	0.97	367	10	59	75	16827	4.09	Calmodulin (CaM)
P06703	1709	−1.51	0.0034	1.69	0.0027	104	2	28	16	10230	5.33	Protein S100-A6
P06703	1723	−1.34	0.004	1.51	1.60E+05	100	2	26	35	10230	5.33	Protein S100-A6
P60709	1773	−1.7	0.00077	−1.44	0.0094	519	14	50	73	41710	5.29	Actin

### Validation of Differentially Modulated Proteins by Western Blotting and Quantitative Real-time PCR in RV Infected HT-29 Cells

**Figure 3:**
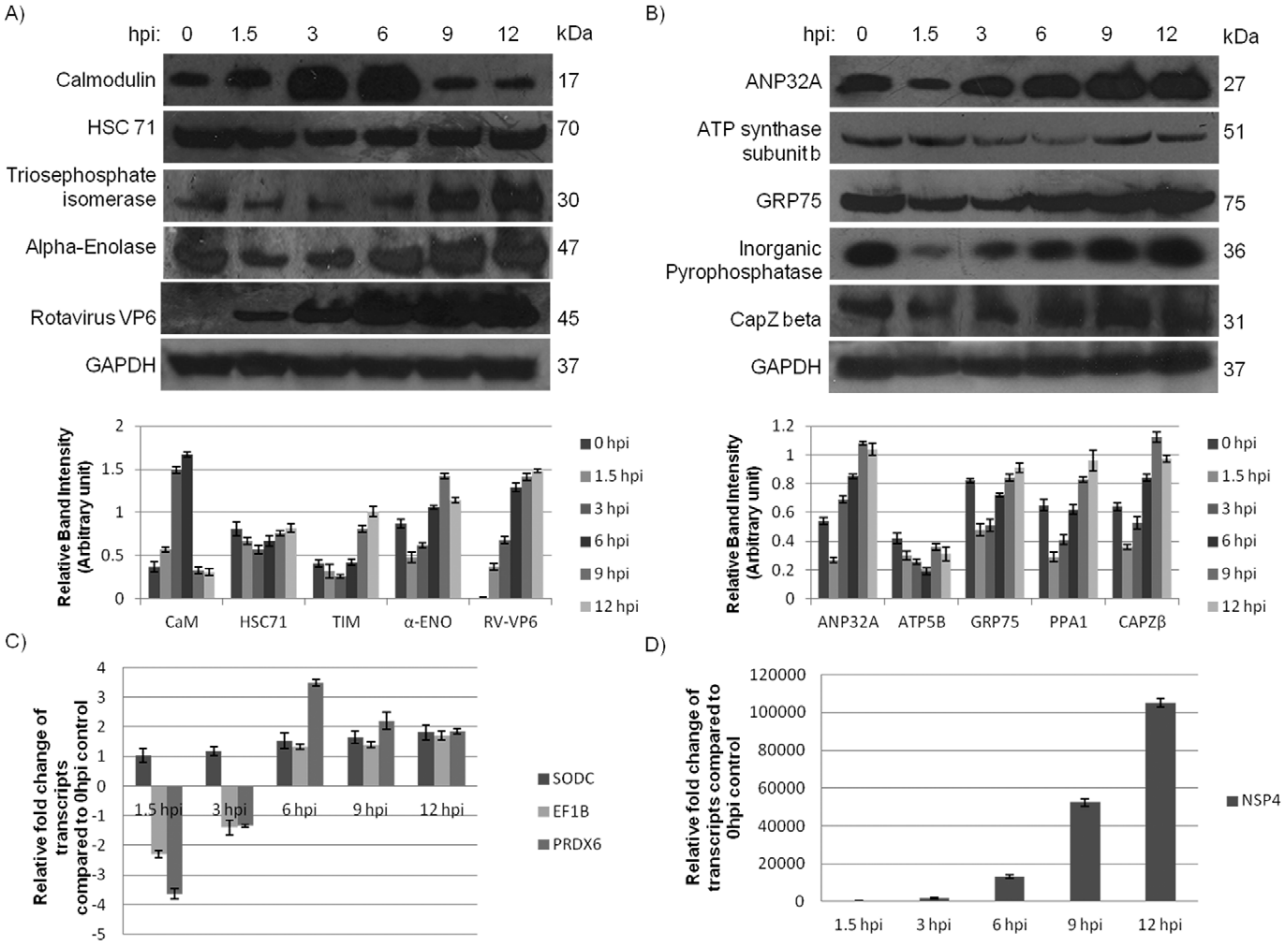
Validation of differentially regulated proteins by immunoblotting and qRT-PCR. **A & B**. Protein were extracted and loaded onto SDS-PAGE from HT 29 cells infected with GARV SA11 strain for indicated time periods followed by Western Blotting. Results shown here are representative of triplicated immunoblotting experiments. **C & D.** 2 µg RNA of SA11 infected (at various time points) HT29 cells were converted to cDNA. 1 µl of each sample were taken for Real-time PCR and amplified with different primers in triplicates. Average value of relative fold change as expressed by 2^−ΔΔCt^ were plotted for each time points. mRNA level of Superoxide dismutase (SODC), elongation factor 1-β (EF1B), peroxiredoxin-6 (PRDX6) and RV-NSP4 are analyzed here.

A total of 12 cellular proteins based on their apparent importance during virus infection or availability of antibodies were validated by western blotting and realtime PCR. These included 9 proteins like heat shock cognate 71 kDa protein, acidic leucine-rich nuclear phosphoprotein 32 family member A, ATP synthase subunit beta, stress-70 protein (GRP 75), alpha-enolase, triosephosphate isomerase, inorganic pyrophosphatase, calmodulin (CaM), F-actin capping protein subunit beta (CapZ beta) were verified by immunoblotting (Fig. 3A & 3B). All 9 proteins showed consistent results with 2D-DIGE based proteomics data (Table 2). Expression of the RV major capsid protein VP6 was assessed to verify virus infection (Fig. 3A). In addition, expression of three genes superoxide dismutase, peroxiredoxin, and enolase was analyzed by quantitative real-time PCR (Fig. 3C) to measure transcript levels. Transcripts of these three genes showed similar trend as proteomics data. RV infection was confirmed by analyzing expression of RV NSP4 mRNA (Fig. 3D).

**Table 2:** Comparison of fold change in 2D-DIGE and Western Blotting of cellular proteins.

Protein Name	Fold change in DIGE		Fold change in Western Blotting in HT29 cells (p<0.05)	
	3 hpi/0 hpi	9 hpi/0 hpi	3 hpi/0 hpi	9 hpi/0 hpi
Calmodulin	1.39	1.00	4.23±0.08	0.99±0.06
HSC71	−1.37	1.11	−(1.43±0.05)	−(1.06±0.08)
Triosephosphate isomerase	−1.16	1.52	−(1.66±0.1)	1.92±0.05
Alpha-Enolase	−1.22	1.53	−(1.34±0.06)	1.7±0.05
Acidic leucine-rich nuclearphosphoprotein 32 familymember A	1.3	2.18	1.19±0.07	2.02±0.06
ATP synthase subunit b	−2.36	1.01	−(1.61±0.05)	1.21±0.08
GRP75	−1.83	−1.2	−(1.65±0.08)	−(1.1±0.07)
Inorganic Pyrophosphatase	−1.18	1.42	−(1.67±0.09)	1.36±0.05
F-actin capping protein subunit beta	−1.29	1.49	−(1.24±0.1)	1.9±0.06

### CaM Protein Colocalized with RV-VP6

**Figure 4:**
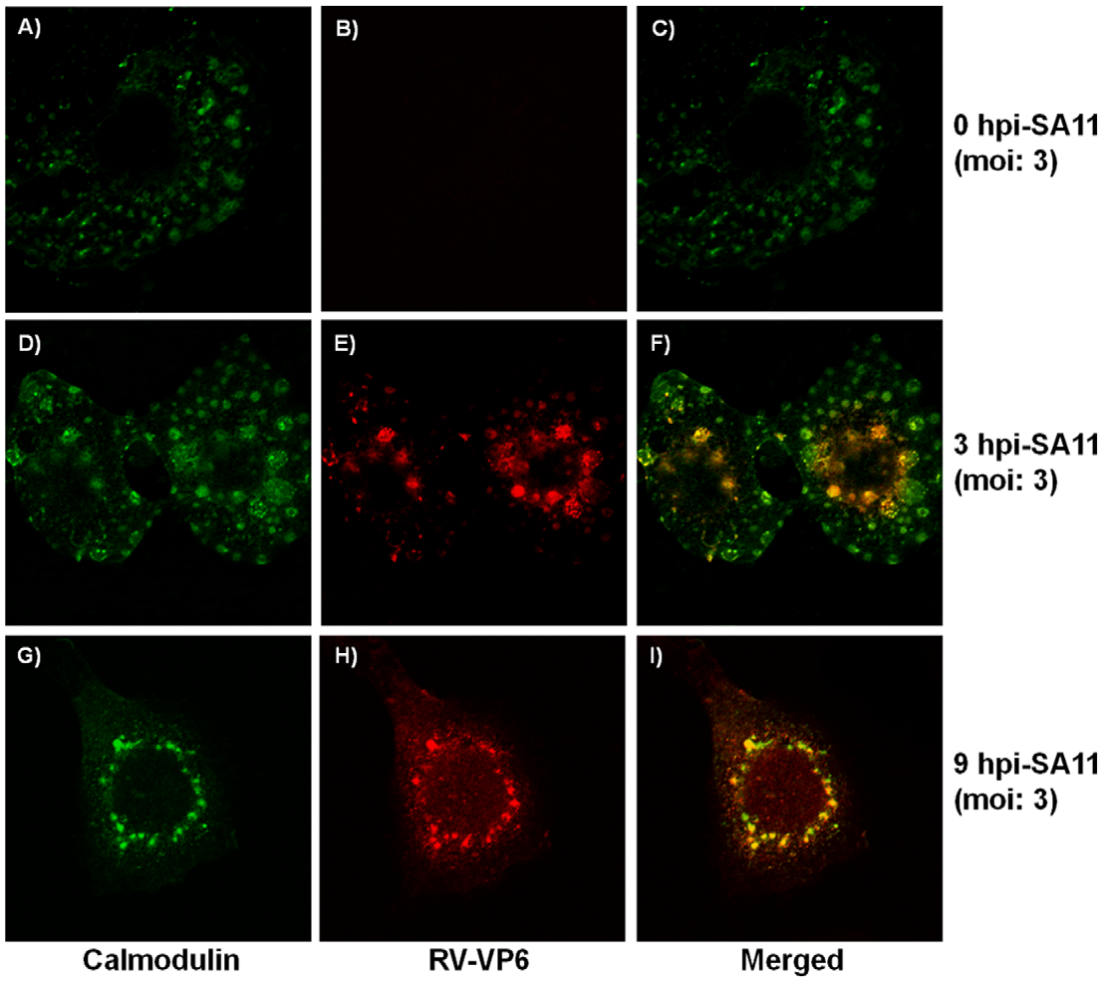
Cellular localization and colocalization pattern of CaM in confocal microscopy. HT29 cells infected for 0 hpi, 3 hpi and 9 hpi were fixed with paraformaldehyde and incubated with CaM and RV VP6 antibody, followed by FITC labeled mouse and Rhodamine labeled rabbit antibodies. High amount of CaM (green) expression was observed at 3 hpi compared to 0 hpi and 9 hpi. Amount of VP6 (red) was higher at 9 hpi than 3 hpi with no expression at 0 hpi. CaM was colocalized with VP6 as evidenced by merged confocal image.

Expression and cellular distribution of CaM was analyzed at 0 hpi, 3 hpi & 9 hpi cells by confocal microscopy. Significantly higher expression of CaM (green) was observed at 3 hpi compared to 9 hpi and 0 hpi (Fig. 4). CaM protein was distributed throughout the cellular cytoplasm. Evenly distributed expression of RV structural protein VP6 (red) in cytoplasm was also observed in an increasing manner from 3 hpi to 9 hpi (Fig. 4). Interestingly, CaM (green) protein colocalized with VP6 (red) (Fig. 4).

### Expression Pattern of CaM during Infection of Different RV Strains

**Figure 5:**
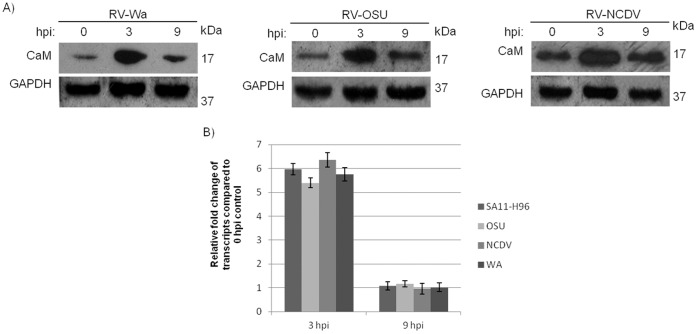
Modulation of CaM in infection of various RV strains. A. Expression of cellular CaM was investigated in infection of Wa, NCDV and OSU strains of RV by immunoblotting at 0 hpi, 3 hpi & 9 hpi. **B.** CaM mRNA level in infection of SA11-H96, Wa, OSU and NCDV strains of RV were analyzed at 3 hpi & 9 hpi.

Expression of cellular CaM was investigated using WA, NCDV and OSU strains of RV at 0, 3 and 9 hpi by immunoblotting and realtime-PCR. All of the three strains showed enhanced level of CaM protein at 3 hpi compared to 0 hpi. At 9 hpi the expression of CaM was comparable to control (Fig. 5A). Similarly Wa, NCDV, OSU and SA11 showed comparable pattern of mRNA in qRT-PCR (Fig. 5B) similar to its protein level.

### Validation of Subset of Differentially Expressed Proteins in BALB/c Mice Model (*in vivo*)

**Figure 6:**
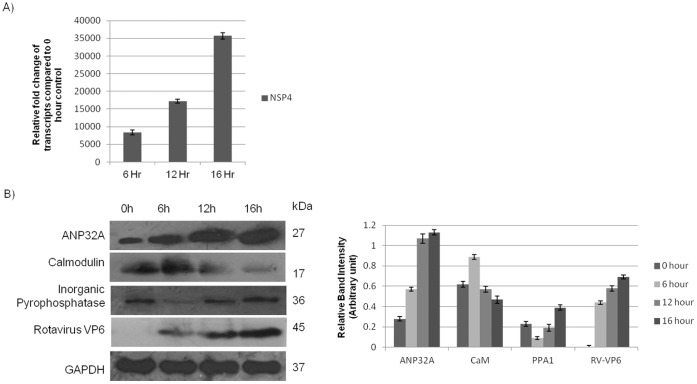
Validation of modulated proteins in *in vivo* model. A. Confirmation of RV infection in mice by qRT-PCR of NSP4 mRNA. **B.** Validation of differentially regulated Proteins in *in-vivo* model. Proteins were extracted from the BALB/c mice infected with GARV SA11 in ligated intestine, 100 µg of protein was loaded onto SDS-PAGE followed by immunoblotting. Results shown here are representative of triplicate experiments. Relative band intensity with respect to GAPDH is expressed as bar graph by densitometric analysis.

To further validate the *in vitro* data in *in vivo* model, expression of a subset of differentially modulated proteins VP6, ANP32A, calmodulin, inorganic pyrophosphatase were tested in mouse intestinal cells following RV infection. BALB/c mice infected with either RV strain SA11 or mock infected were sacrificed at increasing time points. Accumulation of fluid was observed at 6 hr, 12 hr and 16 hr in virus inoculated mice while no such fluid accumulation was detected in mock infected mouse confirming successful infection (data not shown). Virus infection was confirmed by qRT-PCR of extracted RNA from mice by analyzing NSP4 gene of RV (Fig. 6A). Immunoblotting of intestinal protein extract confirmed, level of ANP32A, CaM and Inorganic pyrophosphatase proteins in virus infected sample at different time points and in mock infected control (Fig. 6B). Expression of VP6 protein was assessed by immunoblotting to confirm virus infection in mice (Fig. 6B).

### CaM Protein Interacts with RV VP6 Protein

**Figure 7:**
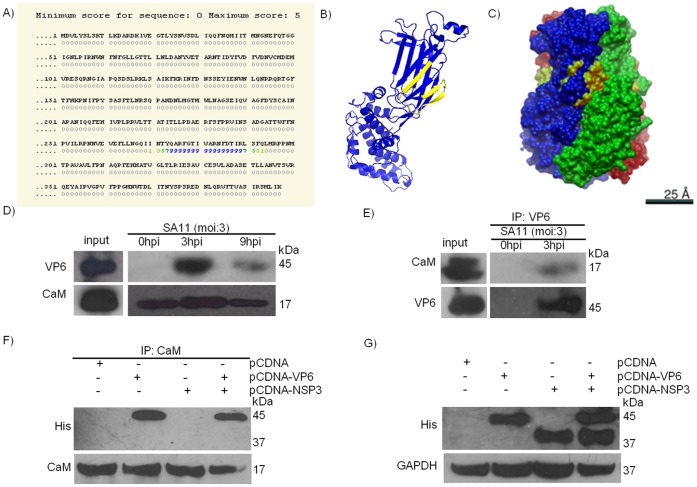
CaM interacts with RV VP6 protein. **A.** Predicted CaM-binding motif was found on RV-VP6 protein with high probability score in Calmodulin target Database. Putative CaM binding motif which spans from 271 to 292 was identified as NTYQARFGTIVARNFDTIRLSF. **B & C.** Position of putative CaM binding site in monomer (**B**) and trimer (**C**) structure of VP6 protein. **D & E.** Protein extract of HT29 cells at 0 hpi, 3 hpi and 9 hpi were incubated with appropriate bead conjugated antibody for overnight followed by detection with another antibody. Data shown here are representatives of three independent experiments. CaM antibody was used to pull down the complex followed by immunoblotting with VP6 (**D**). VP6 antibody was used to immunoprecipitate the complex followed by western blotting with CaM (**E**). **F.** pCDNA-VP6 and pCDNA-NSP3 were transfected individually and together in 293 cells followed by Co-IP with CaM antibody and immunoblotting with His antibody. CaM co-immunoprecipitated with VP6 but not with NSP3. **G.** Expression level of transfected pCDNA-VP6 and pCDNA-NSP3 in 293 cells were assessed by immunoblotting. Data shown in all the segments are representatives of three independent experiments.

As colocalization of CaM protein with RV VP6 was observed in confocal microscopy (Fig. 4), further experiments were carried out. Predicted CaM-binding motif was found on RV VP6 protein with high probability score in Calmodulin target Database. This 22 amino acids motif spans from residues 271 to 292. Putative CaM binding site sequence was identified as NTYQARFGTIVARNFDTIRLSF (Fig. 7A). The putative CaM binding sequence in VP6 monomer and VP6 trimer were shown in 3D model (Fig. 7B & 7C). In monomer, VP6 protein was shown in blue and CaM binding sequence was marked in yellow (Fig. 7B). In trimer structure three monomers were colored in blue, green and red whereas CaM binding site was marked in yellow (Fig. 7B). The CaM binding sequence appears to be surface exposed. To confirm whether VP6 interacts with CaM, Co-IP was performed. Co-IP with CaM specific antibody followed by western blotting with VP6 polyclonal antibody revealed significant amount of VP6 in the immunoprecipitate at 3 hpi, but at 9 hpi interaction was reduced (Fig. 7D). This was consistent with immunofluorescence microscopy results where decreased expression of CaM was observed at 9 hpi. VP6 was not observed in coimmunoprecipitates of 0 hpi infected cell lysates which confirmed specificity of VP6-CAM interaction and of VP6 antibody. Similar results were obtained in reverse experiment where VP6 antibody was used for Co-IP (Fig. 7E). This data was supported by Co-IP of CaM followed by MALDI-TOF/TOF analysis which identifies VP6 as an interactor of CaM with 5 unique peptides match for the specific protein (Figure S2). Transfection of pCDNA-VP6 and pCDNA-NSP3 followed by Co-IP with CaM antibody and immunoblotting with His antibody revealed a 45 kDa protein in pCDNA-VP6 and pCDNA-VP6 & pCDNA-NSP3 transfected samples but not in the pCDNA only and pCDNA-NSP3 transfected samples (Fig. 7F). NSP3 did not co-immunoprecipitate with CaM or VP6. Expression levels of transfected pCDNA-VP6 and pCDNA-NSP3 in 293 cells were confirmed by immunoblotting (Fig. 7G). Overall results confirmed that interaction of VP6 with endogenous CaM is specific.

### CaM Interacts Directly with VP6

**Figure 8:**
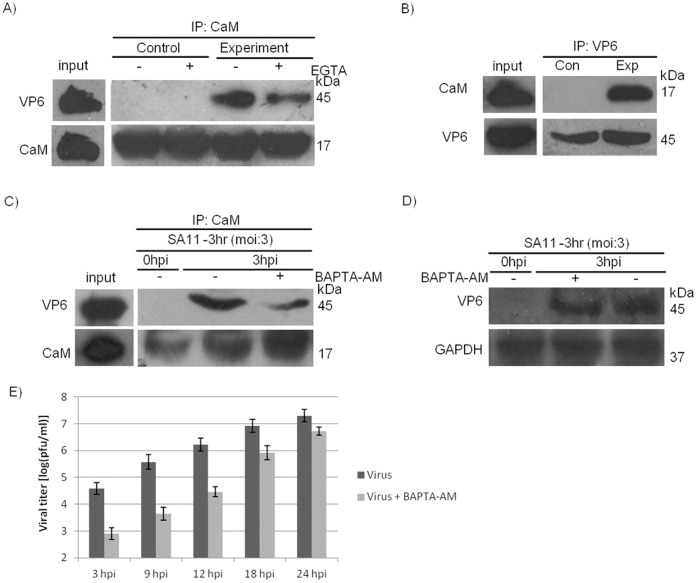
CaM-VP6 interaction is direct and Ca^2+^ dependent. A & B. Affinity purified VP6 and purified CaM were incubated with appropriate bead conjugated antibody for overnight in a CaCl_2_ containing buffer with or without EGTA followed by detection with another antibody. Data shown here are representatives of three independent experiments. CaM antibody was used to pull down the complex followed by immunoblotting with VP6 (**A**). VP6 antibody was used to immunoprecipitate the complex followed by western blotting with CaM (**B**). **C.** Protein extract from HT29 cells at 0 hpi and 3 hpi were incubated overnight with bead conjugated anti-CaM antibody in a Co-IP buffer with or without BAPTA-AM followed by detection with anti-VP6 antibody. CaM level in immunoprecipitant were also shown. Data shown here are representatives of three independent experiments. **D.** Level of VP6 in BAPTA-AM treated and DMSO treated samples at 3 hpi of SA11 infection was analyzed. **E.** HT29 cells were either treated with BAPTA-AM or left untreated and infected for 3 hpi, 9 hpi, 12 hpi, 18 hpi and 24 hpi. Plaque assay were done for all samples, averaged results were expressed as (log (pfu/ml)). Data shown in all the segments are representatives of three independent experiments.

VP6 protein was synthesized using pCDNA-VP6 by *in vitro* coupled transcription and translation and purified with Ni^2+^-NTA magnetic agarose beads. Purified VP6 and CaM proteins were incubated followed by Co-IP with CaM antibody. Immunoblotting revealed precipitation of VP6 with CaM (Fig. 8A). This indicates CaM directly associates with VP6 as there were no other proteins present in the Co-IP mixture. Reciprocal experiments with VP6 antibody confirmed interaction of CaM with VP6 within *in vitro* condition (Fig. 8B).

### VP6-CaM Interaction is Calcium Dependent

CaM interacts with other molecules in either calcium-dependent or calcium-independent manner. Presence of EGTA in Co-IP buffer resulted in reduced interaction between VP6-CaM suggesting that interaction could be Ca^2+^ dependent (Fig. 8A). To confirm cells were either treated with a calcium specific chelator BAPTA-AM prior to infection or treated with DMSO as negative control. In cells treated with BAPTA-AM, reduced amount of VP6 was observed at 3 hpi compared to DMSO treated samples following co-immunoprecipitation with CaM antibody (Fig. 8C). CaM expression levels were found to be same in both BAPTA-AM treated and DMSO treated samples at 3 hpi of SA11 infection (Fig. 8C). VP6 expression was also similar at 3 hpi in presence of BAPTA-AM or DMSO (Fig. 8D). Thus BAPTA-AM (Ca^2+^chelator) treatment does not affect the levels of CaM and VP6 while decreased amount of VP6 was detected in immunoprecipitant treated with BAPTA-AM compared to DMSO treated cells which confirm the calcium dependent interaction of the two molecules. Measurement of PFU in presence and in absence of BAPTA-AM showed significant reduction of viral titers in BAPTA-AM treated cells compared to untreated cells as evidenced by plaque assay expressed as (log (pfu/ml)) (Fig. 8E).

### Ca^2+^/CaM Antagonist W-7 has No Effect on VP6-Ca^2+^/CaM Interaction

**Figure 9:**
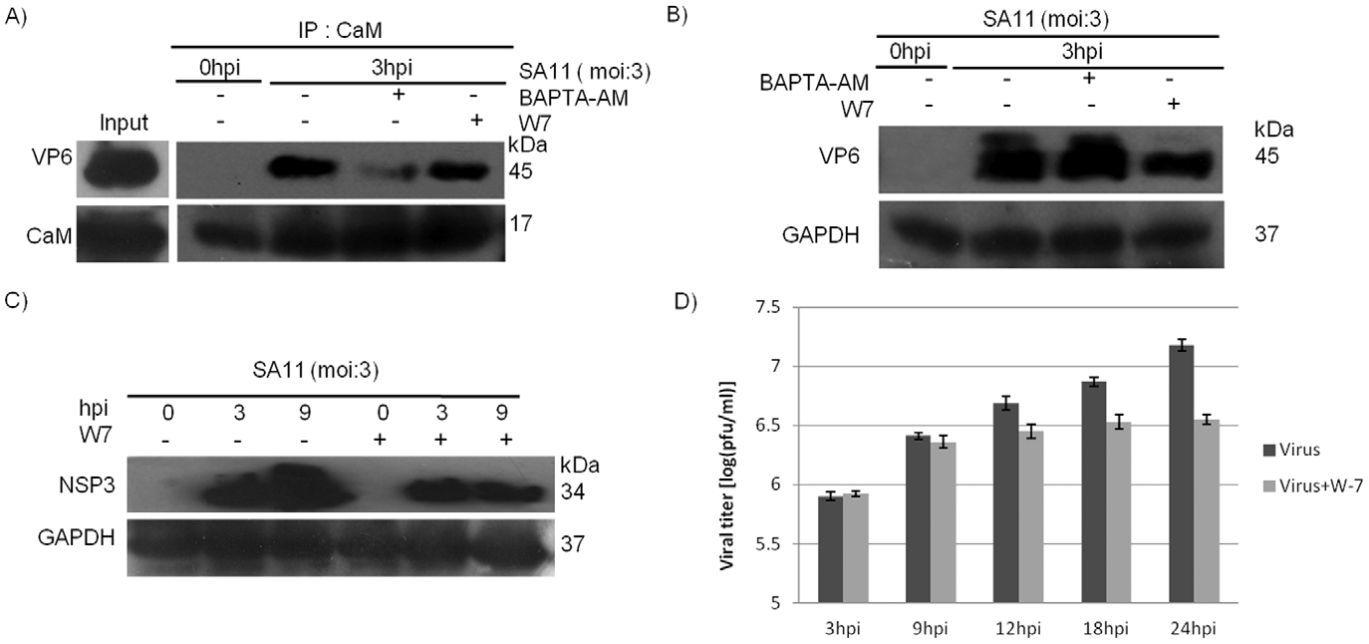
Effect of W-7 on CaM-VP6 association and RV-SA11 titer. **A.** HT29 cells either at 0 hpi or at 3 hpi were immunoprecipitated with CaM antibody. At 3 hpi cells were treated with BAPTA-AM, W-7 and DMSO separately. The immunoprecipitates were probed with VP6 antibody. Another one is left untreated at 0 hpi. **B.** HT29 cells treated with BAPTA-AM, W-7 and DMSO separately were lysed at 3 hpi. Another one is left untreated at 0 hpi. All samples were equally loaded in PAGE and immunoblotted with VP6 antibody. **C.** 0 hpi, 3 hpi and 9 hpi HT29 cells were either treated with W-7 or left untreated. Cells were lysed and immunoblotted with NSP3. **D.** HT29 cells were either treated with W-7 or left untreated and infected for 3 hpi, 9 hpi, 12 hpi, 18 hpi and 24 hpi. Plaque assay were done for all samples, averaged results were expressed as (log (pfu/ml)). Data shown in all the segments are representatives of three independent experiments.

Co-IP was performed on virus infected cells treated with CaM antagonist W-7 or DMSO control, taking CaM as bait and VP6 as prey protein. Results revealed similar levels of VP6 protein in presence or absence of W-7 in Co-IP followed by immunoblotting (Fig. 9A). At 3 hpi, levels of CaM were similar in immunoprecipitates of BAPTA-AM, W-7 or DMSO treated samples (Fig. 9A). Decreased expression of VP6 was observed in W-7 treated cells compared to DMSO control in the input cell lysate (Fig. 9B). Inspite of lower expression of VP6 protein in W-7 treated cells, level of VP6 was similar in W-7 or mock treated immunoprecipitants, suggesting that W-7 does not have any significant effect on VP6-Ca^2+^/CaM interaction.

### Ca^2+^/CaM Antagonist W-7 Results in Reduced Expression of RV Encoded Proteins and Decrease in Viral Titer

Since W-7 affected VP6 expression, its effect on other RV encoded proteins was measured. In W-7 treated cells significant decrease in expression of NSP3 protein was observed at 3 hpi & 9 hpi (Fig. 9C). Negative effect of W-7 on RV was also confirmed when viral titers were measured in presence or absence of W-7. The amount of virus particles were significantly reduced in W-7 treated cells compared to untreated cells as evidenced by plaque assay expressed as (log (pfu/ml)). During early hours of infection (≤9 hpi), effect of W-7 on viral titer was not significant but as the viral life cycle progressed, considerable decrease in viral titer was observed at 24 hpi in W-7 infected cells compared to DMSO control (Fig. 9D). This effect may be due to antagonistic activity of the W-7 against the Ca^2+^-CaM complex [36] which is required for efficient viral replication & assembly.

## Discussion

The studies of the rotaviruses are mostly confined to either analysis of specific function of RV encoded proteins or epidemiology, or vaccine development. The RV induced alteration of host cell proteome was not studied extensively, until recently when Zambrano *et. al.* reported RV induced changes of cellular proteome in the context of IFN response [16]. The results led to the identification of proteins related to the cell stress mediated by RV OSU strain in presence or in absence of IFN treatment. GRP78 and GRP 94 protein which were upregulated during RRV infection were surprisingly found to be downregulated in OSU infected cells reflecting differences among various RV strains [16]. This was consistent with previous observations that degradation of IRF3 by RV varies among different strains and cell lines [37]. In the current study, an attempt has been made to identify host cellular proteins with altered expression level at early (3 hpi) and late (9 hpi) phase of RV infection using SA11 strain. 2D-DIGE analysis followed by mass spectrometry led to the identification of 22 unique proteins that had differential protein expression. Among the 22 unique proteins identified by MALDI-TOF/TOF MS, expression of 9 genes was validated by western blotting and 3 were validated by Real-time PCR. These were further confirmed using *in vivo* animal model. Though ligated intestinal loops in colostrum-deprived calves have been used previously to evaluate the effect of four isolates of bovine rotavirus, it has not been tested in other experimental animals like rabbit, mice etc. [38]. An intestinal ligated loop model for RV infection in BALB/c mouse was established here as reported previously for *Vibrio cholerae* [27] and *Staphylococcus aureus* [28]. Accumulation of fluid and upregulation of NSP4 transcript in ligated loop of RV infected mice confirmed virus infection in BALB/c model. Expression of ANP32A, CaM and inorganic pyrophosphatase proteins *in vivo* were comparable to *in vitro* data. As shown in Figure 3A, 4 & 5A, CaM was upregulated at 3 hpi but at 9 hpi, level of CaM expression was similar to uninfected control. Analysis of CaM expression by confocal microscopy confirmed increased CaM expression in RV infected cells (3 hpi) (Fig. 4), surprisingly CaM (red) colocalized with RV protein VP6 (green) suggesting possible interaction.

CaM is a constitutively expressed 12 kDa Ca^2+^ binding protein which upregulated in rapidly growing cells or cells reaching G1/S boundary [39]. CaM levels are also reciprocally regulated by Ca^2+^ concentration [39], [40], [41]. CaM is a calcium binding protein of EF-hand family, which is found in both Ca^2+^ free and Ca^2+^ bound states [39], [42]. One of the main functions exhibited by Ca^2+^/CaM and its targets is regulation of cell cycle progression but not much is known about the mechanism. CaM is a very promiscuous molecule due to its structural flexibility and highly adaptable nature of the binding surface [43]–[46]. CaM contains four EF-hand motifs which can bind four Ca^2+^ ions, resulting in huge structural shift of CaM from the unbounded state which enables binding of large number of proteins collectively termed CaM Binding Proteins [42], [47]. Ca^2+^/CaM have various downstream targets like serine/threonine phosphatase, calcineurin, multifunctional Ca^2+^/CaM -dependent protein kinases (CaM kinase II), adenyl cyclases, ion channels, phosphodiesterases, myosin light chain kinases and protein phosphatases [39]. Ca^2+^/CaM is also known to significantly enhance PI3-Kinase activity *in vitro* [48]. Increased CaM causes differences in the organization of microfilaments, intermediate filaments, & microtubules; these changes are accompanied by differences in the cell-cycle dependent expression of some mRNAs [49]. For efficient viral replication, viruses such as EBV, HIV-1 induce cell cycle transition from G1 to S phase [50], [51]. So it is possible that during early RV infection (3 hpi) cell cycle progresses from G1 to S phase. Previous studies have shown that translation machinery and other cellular system are highly active during S phase which may benefit viruses as they can efficiently synthesize their proteins using host cell components. Activation of PI3Kinase/AKT during early hours of infection (2 hpi) also correlates with increased expression of CaM which is known to enhance PI3Kinase activity [17], [48]. Recently novel role of Ca^2+^/CaM in RV life cycle has been discovered where, by inducing Ca^2+^/CaM dependent kinase kinase β signaling, virus hijacks the autophagy membrane trafficking pathway for transporting viral proteins to sites of virus replication [52]. Overall the results suggest upregulation of CaM has significance during RV infection.

VP6 of RV is a major structural protein of the virus consisting of majority of the viral capsid [53]. This protein is highly conserved among various strains and highly immunogenic in nature [54], [55] and is also the basis of group and subgroup classification of the virus [56], [57]. The VP6 polypeptide is folded into two distinct domains, Domain B consists of eight α-helices with amino acids from two separate regions (1–150 & 331–397) and Domain H contains residues 151–331 making a β-sandwich structure [53]. The T-helper cells recognize epitopes on VP6 resulting in antiviral polyclonal T-helper cell response in spleen [58]. VP6 also interacts with B lymphocytes in healthy RV-exposed adults, RV-infected infants, and RV-naive neonates [59]. Cytotoxic T-cell epitope were also identified in VP6 protein of rotavirus [60]. When mice were immunized with a chimeric VP6 protein, IFN-gamma was the only anti-rotavirus cytokine found after *in vitro* stimulation of memory CD4+ T cells [61]. Three of the four llama-derived single chain antibody fragments directed towards VP6 protein possess broad neutralizing activity *in vitro* and gave protection against diarrhea in mice [62]. In addition monoclonal antibodies against VP6 have been shown to inhibit *in vitro* transcription of rotavirus, indicating role of RV-VP6 in viral transcription [63]. Recently, VP6 has been found to interact with cellular SUMOylation system and gets post-translationally modified with SUMO [64]. In spite of these studies, relatively less information is available to date, in the realm of cellular effects of VP6.

Consistent with confocal microscopy results, VP6-CaM interaction was confirmed by Co-IP experiment following RV infection (Fig. 7D & E) as well as by using purified CaM protein and IVT VP6 in cell free system (Fig. 8A & B). Significantly, the interaction of VP6 and CaM in cell-free system where no other cellular proteins were present confirmed direct binding of CaM with VP6 protein (Fig. 8A & B). Sequence analysis of VP6 also revealed putative CaM binding site (Fig. 7A). CaM may have significant role during RV infection since it binds to Ca^2+^ [39], [42] and role of RV in disruption of Ca^2+^ homeostasis during infection which alters cytoskeletal proteins is well documented [33], [34], [65]. To confirm whether VP6-CaM interaction was Ca^2+^ dependent or Ca^2+^ independent, cell permeable Ca^2+^ specific chelator BAPTA-AM was used since it had no effect on VP6 expression (Fig. 8D). As shown in Fig. 8C, VP6-CaM interaction was Ca^2+^ dependent thus it was referred as Ca^2+^/CaM-VP6 interaction. In addition Ca^2+^ chelator EGTA in Co-IP buffer also disrupted CaM-VP6 binding (Fig. 8A). In contrast, W-7, a CaM antagonist which has been known to bind calmodulin selectively to inhibit Ca^2+^-CaM dependent enzyme activities, cell proliferation and G1-S transition [66] had no effect on VP6-CaM interaction, though it resulted in downregulation of VP6 (Fig. 9A & B).

In addition to VP6, W-7 treatment also resulted in decreased expression of another RV encoded protein NSP3 suggesting overall inhibition of rotavirus replication process at either transcription or translation level (Fig. 9C). Ca^2+^/CaM positively modulate RV infection as significant decrease in viral titer was observed in presence of W-7 (Fig. 9D). Detailed investigation of the specific mode of action of the W-7 is required to understand the underlying phenomena to understand Ca^2+^/CaM role in RV pathogenesis as well as to develop W-7 or other CaM inhibitors as anti-rotavirals in future.

### Conclusion

This study not only sheds light on the RV mediated proteome change in HT-29 cells, but also shows positive involvement of cellular Ca^2+^ binding protein CaM during viral pathogenesis. In summary, it can be stated that CaM along with other cellular proteins are differentially regulated as evident from the 2D-DIGE based proteomics study and CaM positively regulates RV infection. In-depth analysis of other differentially modulated proteins is required to understand their role during virus infection.

## Supporting Information

Figure S1 **Infection of RV-SA11 at different moi/time.** RV-SA11 was infected in HT-29 cells at 1, 3 and 5 moi for 0, 3 and 9 hpi. Cells were stained with anti-NSP5 primary antibody followed by FITC conjugated secondary antibody; nucleus was stained by DAPI and observed under fluorescence microscope.
(TIF)

Figure S2 **Proteomic level confirmation of CaM-VP6 interaction.** A. Co-IP of 0 hpi and 3 hpi samples were done using CaM antibody. SDS-PAGE analysis of the immunoprecipitates show suspected band of VP6 at around 45 kDa. B. MALDI-TOF/TOF analysis of the suspected band resulted in matching of 5 peptides with that of VP6.
(TIF)

Text S1 **Appendix.**
(DOCX)
