# Supplementary figures and images for: Identification of Cellular Calcium Binding Protein Calmodulin as a Regulator of Rotavirus A Infection during Comparative Proteomic Study
Source: PLoS One. 2013 Feb 20;8(2):e56655. doi: 10.1371/journal.pone.0056655 (PMC3577757; doi:10.1371/journal.pone.0056655)

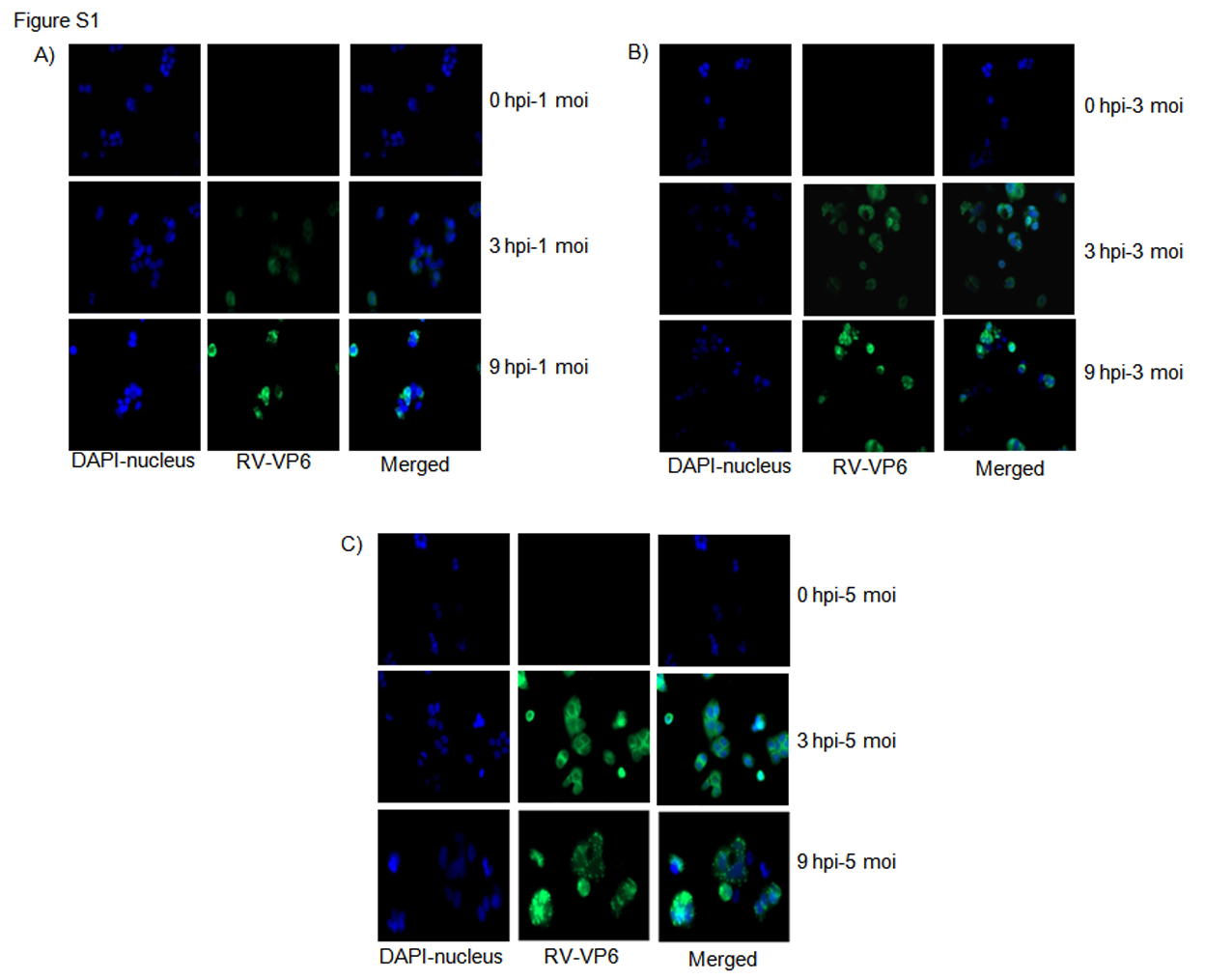

Supplement: Figure S1 — Infection of RV-SA11 at different moi/time. RV-SA11 was infected in HT-29 cells at 1, 3 and 5 moi for 0, 3 and 9 hpi. Cells were stained with anti-NSP5 primary antibody followed by FITC conjugated secondary antibody; nucleus was stained by DAPI and observed under fluorescence microscope. (TIF) [file pone.0056655.s001.tif]

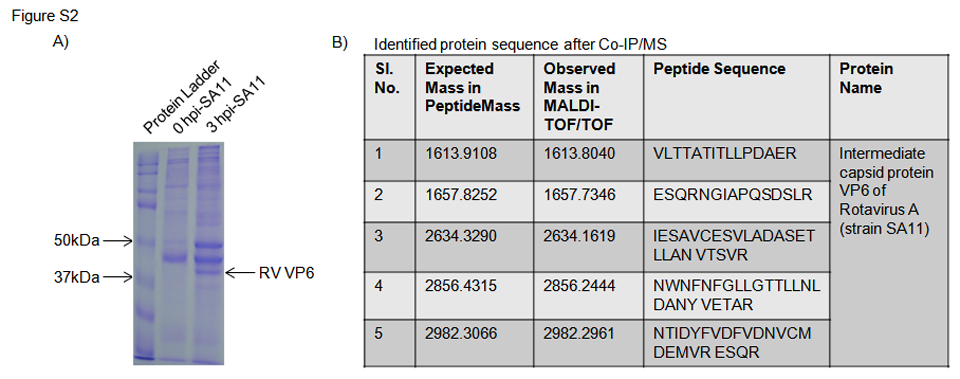

Supplement: Figure S2 — Proteomic level confirmation of CaM-VP6 interaction. A. Co-IP of 0 hpi and 3 hpi samples were done using CaM antibody. SDS-PAGE analysis of the immunoprecipitates show suspected band of VP6 at around 45 kDa. B. MALDI-TOF/TOF analysis of the suspected band resulted in matching of 5 peptides with that of VP6. (TIF) [file pone.0056655.s002.tif]
