# Supplementary material for: Identification of Cellular Calcium Binding Protein Calmodulin as a Regulator of Rotavirus A Infection during Comparative Proteomic Study
Source: PLoS One. 2013 Feb 20;8(2):e56655. doi: 10.1371/journal.pone.0056655 (PMC3577757; doi:10.1371/journal.pone.0056655)
Supplement: Text S1 — Appendix. (DOCX) [file pone.0056655.s003.docx]

Appendix

GARV, Group A Rotavirus; CaM, Calmodulin; Co-IP, Co-immunoprecipitation; 2D-DIGE, Two-dimensional Difference Gel Electrophoresis.
